# Supplementary material for: Role of aspirin on colorectal cancer risk and bacterial translocation to bloodstream
Source: PLoS One. 2025 Mar 28;20(3):e0319750. doi: 10.1371/journal.pone.0319750 (PMC11952268; doi:10.1371/journal.pone.0319750)

**S1 Table.** P for tests comparing relative abundances of bacterial taxa and OTUs and the odds ratios (OR) and the corresponding 95% confidence intervals (CI) of the presence of these bacteria for aspirin use. Italy 2017-2019.

| **Bacterial**  **taxa/**  **OTUs** | **Taxonomy; regular aspirin use versus non use** | **p-value**  **Wilcoxon 2-sided test** | **p-value**  **Chi-squared test** | **OR^a^ (CI)** | **OR^b^ (CI)** |
| --- | --- | --- | --- | --- | --- |
| Order | Bacteria;Actinomycetota;Acidimicrobiia;Microtrichales o. | 0.04 | 0.04 | 2.45 (1.03-5.82) | 2.49 (1.04-5.93) |
| OTU _181 | Bacteria;Actinomycetota;Actinomycetes;Corynebacteriales;Corynebacteriaceae;Corynebacterium 1;Multi-affiliation | 0.05 | 0.04 | 2.65 (0.99-7.07) | 2.74 (1.02-7.38) |
| Order | Bacteria;Actinomycetota;Actinomycetes;Propionibacteriales o. | 0.02 | 0.26 | 0.57 (0.21-1.54) | 0.57 (0.21-1.54) |
| Family | Bacteria;Actinomycetota;Actinomycetes;Propionibacteriales;Propionibacteriaceae f. | 0.01 | 0.32 | 0.61 (0.23-1.64) | 0.61 (0.23-1.64) |
| Genus | Bacteria;Actinomycetota;Actinomycetes;Propionibacteriales;Propionibacteriaceae;Cutibacterium g. | 0.009 | 0.32 | 0.61 (0.23-1.64) | 0.61 (0.23-1.64) |
| OTU_343 | Bacteria;Actinomycetota;Actinomycetes;Propionibacteriales;Propionibacteriaceae;Cutibacterium;Multi-affiliation | 0.009 | 0.32 | 0.61 (0.23-1.64) | 0.61 (0.23-1.64) |
| Order | Bacteria;Actinomycetota;Thermoleophilia;Gaiellales;Gaiellales o. | 0.05 | 0.04 | 2.65 (0.99-7.07) | 2.67 (1.00-7.14) |
| Family | Bacteria;Actinomycetota;Thermoleophilia;Gaiellales;Gaiellaceae f. | 0.02 | 0.02 | 3.40 (1.13-10.22) | 3.49 (1.15-10.54) |
| Genus | Bacteria;Actinomycetota;Thermoleophilia;Gaiellales;Gaiellaceae;Gaiella g. | 0.02 | 0.02 | 3.40 (1.13-10.22) | 3.49 (1.15-10.54) |
| Genus | Bacteria;Firmicutes;Clostridia;Clostridiales;Peptostreptococcaceae; Romboutsia g. | 0.04 | 0.04 | 0.24 (0.06-1.05) | 0.25 (0.06-1.05) |
| Family | Bacteria;Proteobacteria;Alphaproteobacteria;Rickettsiales;Mitochondria f. ^c^ | 0.02 | 0.02 | - | - |
| Order | Bacteria;Proteobacteria;Alphaproteobacteria;Sphingomonadales o. | 0.03 | 0.08 | 0.41 (0.15-1.16) | 0.40 (0.14-1.14) |
| Family | Bacteria;Proteobacteria;Alphaproteobacteria;Sphingomonadales;Sphingomonadaceae f. | 0.03 | 0.08 | 0.41 (0.15-1.16) | 0.40 (0.14-1.14) |
| Genus | Bacteria;Proteobacteria;Alphaproteobacteria;Sphingomonadales;Sphingomonadaceae;Sphingomonas g. | 0.01 | 0.32 | 0.61 (0.23-1.64) | 0.60 (0.22-1.61) |
| OTU_144 | Bacteria;Proteobacteria;Alphaproteobacteria;Sphingomonadales;Sphingomonadaceae;Sphingomonas;Multi-affiliation | 0.02 | 0.16 | 0.58 (0.27-1.24) | 0.58 (0.27-1.24) |
| Class | Bacteria;Proteobacteria;Deltaproteobacteria c. | 0.03 | 0.04 | 0.55 (0.31-0.99) | 0.55 (0.31-0.99) |
| Genus | Bacteria;Proteobacteria;Gammaproteobacteria;Betaproteobacteriales;Burkholderiaceae;Delftia g. | 0.03 | 0.03 | 2.05 (1.06-3.97) | 2.07 (1.06-4.03) |
| OTU_39 | Bacteria;Proteobacteria;Gammaproteobacteria;Betaproteobacteriales;Burkholderiaceae;Delftia;Multi-affiliation | 0.03 | 0.03 | 2.23 (1.07-4.64) | 2.23 (1.07-4.64) |

^a^Estimated from logistic regression models.

^b^Estimated from logistic regression models adjusted for a term of controls/intestinal adenoma/colorectal cancer.
^c^Taxa absent in aspirin users

**S1 Figure.** Flow chart of data collection procedure.


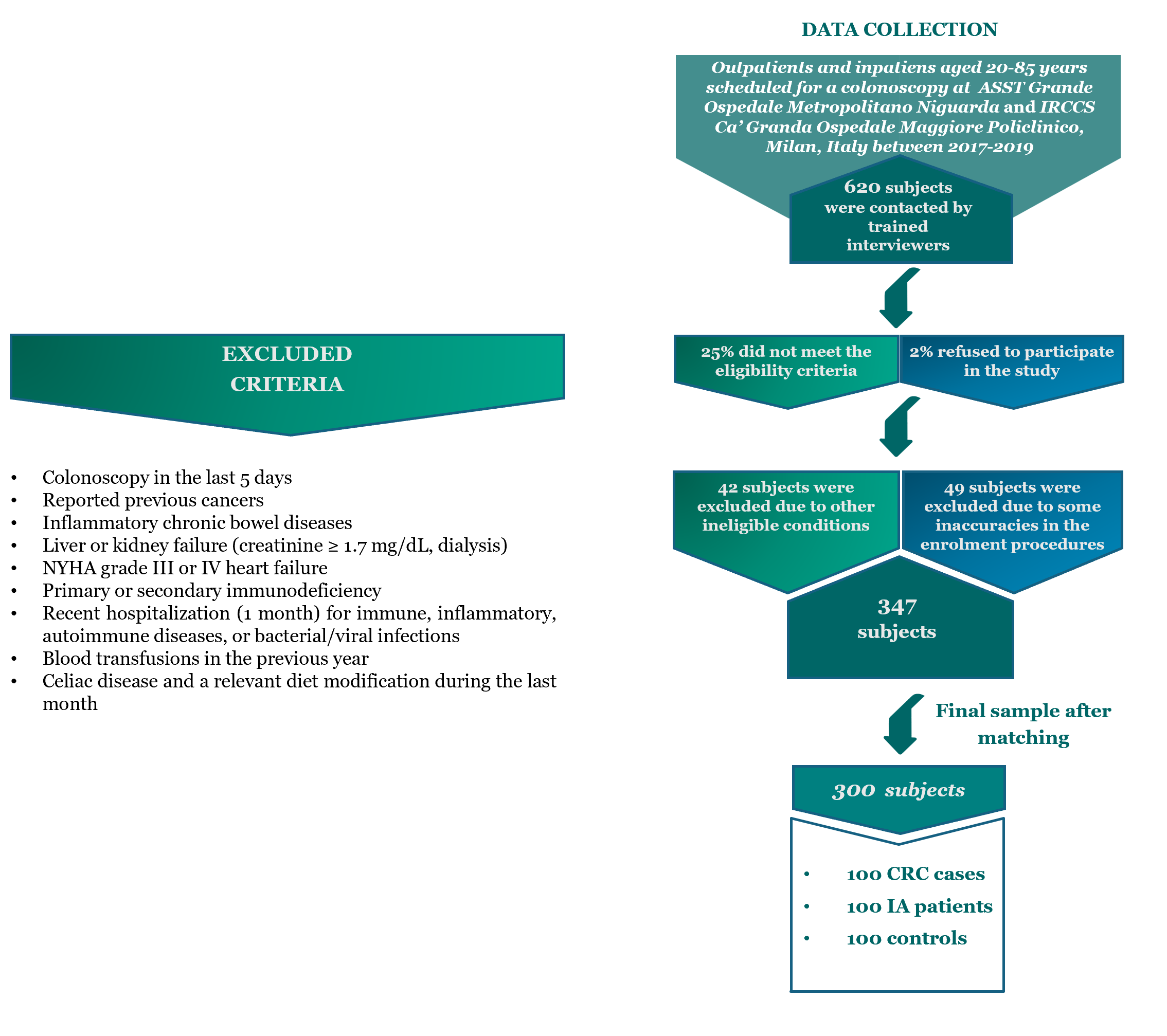

Supplement: S1 File — S1 Fig. Flow chart of data collection procedure; S1 Table. P for tests comparing relative abundances of bacterial taxa and OTUs and the odds ratios (OR) and the corresponding 95% confidence intervals (CI) of the presence of these bacteria for aspirin use. Italy 2017-2019. (DOCX) [file pone.0319750.s001.docx]
